# Mediastinal mature teratoma with chest pain onset and subsequent perforation: A case report

**DOI:** 10.1016/j.ijscr.2021.105807

**Published:** 2021-03-26

**Authors:** Tsuyoshi Uchida, Hirochika Matsubara, Tamami Hada, Daisuke Sato, Norio Hasuda, Hiroyuki Nakajima

**Affiliations:** Department of Thoracic Surgery, Yamanashi University, 1110 Shimkato, Chuo-shi, Yamanashi, Japan

**Keywords:** CT, computed tomography, MRI, magnetic resonance imaging, Mediastinal mature teratoma, Chest pain, Perforation, Emergency, Paediatric surgery, Thymectomy

## Abstract

**Introduction:**

Mediastinal mature teratomas are often benign, asymptomatic, and incidentally detected during routine chest roentgenography. Enzymes secreted by intestinal or pancreatic tissue in teratomas may lead to mediastinitis or the rupture of adjacent tissues. Herein, we present a case of a patient who experienced sudden onset of chest pain followed by the perforation of a mediastinal teratoma.

**Presentation of case:**

A 10-year-old boy presented with chest pain 2 days before admittance to the hospital. Chest radiography showed an anomalous mass shadow, and computed tomography showed an anterior mediastinal mass. Radiography revealed an increase in the mass shadow size and dullness of the left costal phrenic angle. Magnetic resonance imaging revealed pleural effusion and intratumoral haemorrhage, indicating perforation of the tumour. Emergency excision and thymectomy via sternotomy were performed. Pathology confirmed that the mediastinal tumour presented no immature or malignant elements.

**Discussion:**

In the present case, the onset of chest pain occurred 2 days before admission, and the initial computed tomography did not reveal tumour perforation. Subsequent chest radiography and magnetic resonance imaging indicated that the tumour had perforated. Surgical tumour excision was planned at the time of admission; however, once perforation was confirmed, emergency surgery was performed. The pleural effusion had high cancer antigen 19-9 levels, and this was expected as the pleural effusion contained pancreatic digestive enzymes.

**Conclusion:**

The perforation of a mediastinal mature teratoma cannot be predicted based on the symptoms, tumour size, or onset of pain alone. Once perforation is confirmed, surgical excision should be performed immediately.

## Highlights

• Ruptured mediastinal teratomas (RMTs) may lead to mediastinitis.
• RMTs may also cause the rupture of adjacent tissues.
• Immediate resection of RMTs should be performed once perforation is confirmed.
• Radiography and MRI may provide useful information for RMT diagnosis.

## 1 Introduction

Mediastinal mature teratomas are often benign and asymptomatic [[1], [2], [3], [4], [5], [6], [7]]. These tumours are either incidentally detected during routine chest roentgenography or cause symptoms such as wheezing or dysphagia resulting from the compression of adjacent structures. Digestive enzymes secreted by the intestinal mucosa or pancreatic tissue found in teratomas may lead to the rupture of the bronchi, pleura, pericardium, or lungs [1]. Additionally, prior to rupture, these enzymes may cause mediastinitis. Thus, it is recommended that resection of the teratoma be performed as soon as possible [2]. However, for a safe resection, an evaluation through imaging should be conducted. Herein, we present a case of a teratoma in a patient who experienced a sudden onset of chest pain followed by tumour perforation that was observed during a closer examination. The present case report is in compliance with the SCARE guidelines [3]. Informed consent was obtained from the patient and the patient’s parents.

## 2 Presentation of case

**Fig. 1:**
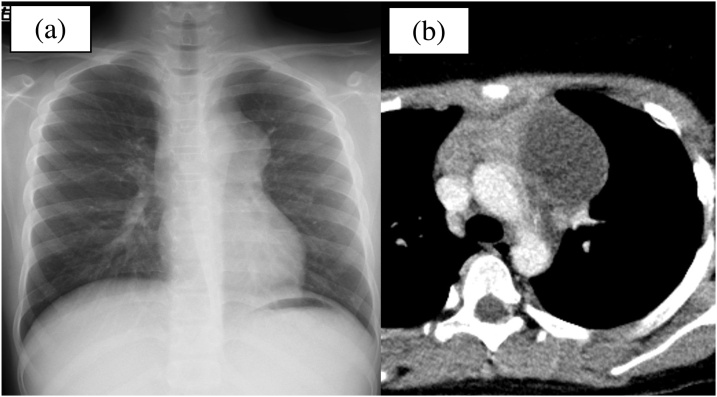
Patient images on the day of admission. (a) Chest radiography showing a large mediastinal mass. (b) Computed tomography showing a cystic mass with solid components in the anterior mediastinum.

**Fig. 2:**
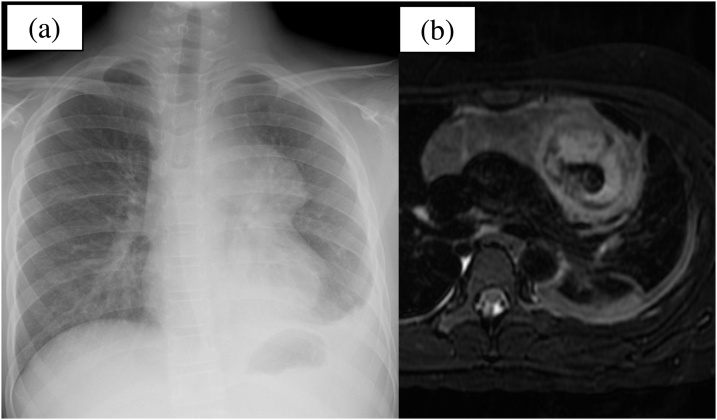
Patient images on the second day of admission. (a) Chest radiography showing that the tumour’s shadow has increased and the left costal phrenic angle is dull. (b) Magnetic resonance imaging revealing pleural effusion and intratumoral haemorrhage.

A 10-year-old boy with no medical history who was experiencing chest pain for 2 days was admitted to our hospital. Chest radiography showed a mass shadow in contact with the aortic shadow, and computed tomography (CT) showed an anterior mediastinal tumour (Fig. 1). Blood test results revealed an elevated white blood cell count of 12,550/μL and C-reactive protein levels of 8.6 mg/L. The tumour was suggested to be a mature cystic teratoma with an inflammatory reaction. The day following admission, a chest radiography showed that the mass shadow had increased in size and that the left costal phrenic angle was dull. Furthermore, magnetic resonance imaging (MRI) revealed pleural effusion and intratumoral haemorrhage (Fig. 2). Consequently, intrathoracic perforation was suggested, and emergency mediastinal tumour excision and thymectomy via sternotomy were performed.

Once the sternotomy was performed, the left thoracic cavity where the tumour was protruding was intentionally opened. There was a moderate volume of viscous pleural effusion. The tumour was involved with the left phrenic nerve and showed adhesions to the thymus. Tumour excision and thymectomy were successfully performed, and the left phrenic nerve was detached from the tumour using Metzenbaum scissors. The thoracic cavity and mediastinum were irrigated with 5,000 mL of saline solution after the excision.

**Fig. 3:**
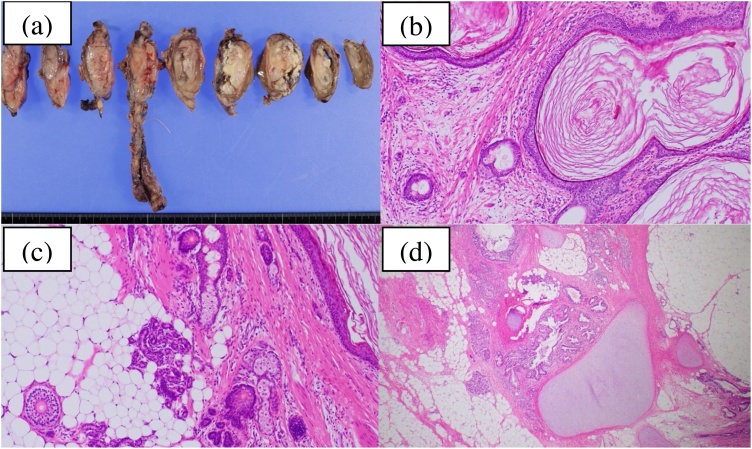
Pathological findings of the resected teratoma. (a) Cut surface of the resected mature teratoma with solid and cystic components. Histological findings show a mature teratoma with multiple organ components. (b) Stratified squamous epithelium and keratinised tissue. (c) Pancreatic tissue, including acinar cells and ductal elements. (d) Bronchi and bronchial cartilage.

The patient’s recovery was uneventful. His chest tube was removed on the fifth postoperative day, and he was discharged the following day. Phrenic nerve palsy was not observed. Pathology confirmed that the mediastinal tumour was a mature teratoma with no immature or malignant elements (Fig. 3).

## 3 Discussion

Reportedly, 50–62 % of mature teratomas are asymptomatic [4,5]. In contrast, 35–41 % of patients have been reported to have perforation-related symptoms such as chest pain and fever [6,7]. In this case report, because the onset of chest pain occurred 2 days before admission, the initial CT did not reveal that the tumour was perforated. However, on the day after the admission, chest radiography and MRI indicated that the tumour had perforated. Therefore, timely MRI for preoperative diagnosis and follow-up radiography resulted in the early detection of the perforated tumour.

Cystic teratomas may perforate adjacent organs but tumour diameter and wall thickness are not associated with the risk of perforation [7]. The effusion of a mature teratoma contains digestive enzymes that cause inflammation and adhesion. Therefore, surgical management is often more complicated for perforated tumours than for unperforated ones. Hence, cystic teratomas should be resected immediately after diagnosis [2]. In the present case, we planned the surgical tumour excision at the time of admission; however, once perforation was confirmed, emergency surgery was performed. The pleural effusion observed during the surgery showed high cancer antigen 19-9 levels, which was expected because the pleural effusion contained pancreatic digestive enzymes. However, early surgery did not cause any postoperative problems except for mild adhesions and inflammation.

Therefore, it is important to perform surgical excision immediately after confirming tumour perforation.

## 4 Conclusion

We identified two important clinical issues in the present case. First, the perforation of a mediastinal mature teratoma cannot be predicted solely based on the symptoms, tumour size, and timing of onset. Second, it is important to perform immediate surgical excision once the tumour has been perforated. The present case demonstrates the importance of early diagnosis of a ruptured mediastinal teratoma using radiography and MRI. Though a ruptured mediastinal teratoma is not particularly rare, it is rare to locate images of a teratoma before and after the rupture. However, our case report successfully captured these images.

## Declaration of Competing Interest

The authors report no declarations of interest.

## Sources of funding

I have no sources of funding should be declared.

## Ethical approval

Our institutional review board gave echical exemption for this study.

## Consent

Written informed consent was obtained from the patient for publication of this case report and accompanying images. A copy of the written consent is available for review by the Editor-in-Chief of this journal on request.

## Author contribution

**Tsuyoshi Uchida:** Writing – original draft, Data curation, Formal analysis.

**Hirochika Matsubara:** Writing – review & editing.

**Tamami Hada:** Writing – review & editing, Data curation, Formal analysis.

**Daisuke Sato:** Writing – review & editing, Data curation, Formal analysis, Conceptualization.

**Norio Hasuda:** Writing – review & editing.

**Hiroyuki Nakajima:** Writing – review & editing, Supervision.

## Registration of research studies

Not applicable.

## Guarantor

Tsuyoshi Uchida.

## Provenance and peer review

Not commissioned, externally peer-reviewed.

## Data statement

The datasets generated and analysed during the current study are not publicly available as this is a case report, but they are available from the corresponding author on reasonable request.
